# TAR Cloning: Perspectives for Functional Genomics, Biomedicine, and Biotechnology

**DOI:** 10.1016/j.omtm.2019.05.006

**Published:** 2019-05-21

**Authors:** Natalay Kouprina, Vladimir Larionov

**Affiliations:** 1 Developmental Therapeutics Branch, National Cancer Institute, Bethesda, MD 20892, USA

**Keywords:** transformation-associated recombination, TAR cloning, gene functional studies, human artificial chromosome, HAC, gene delivery vector, CRISPR/Cas9

## Abstract

Completion of the human genome sequence and recent advances in engineering technologies have enabled an unprecedented level of understanding of DNA variations and their contribution to human diseases and cellular functions. However, in some cases, long-read sequencing technologies do not allow determination of the genomic region carrying a specific mutation (e.g., a mutation located in large segmental duplications). Transformation-associated recombination (TAR) cloning allows selective, most accurate, efficient, and rapid isolation of a given genomic fragment or a full-length gene from simple and complex genomes. Moreover, this method is the only way to simultaneously isolate the same genomic region from multiple individuals. As such, TAR technology is currently in a leading position to create a library of the individual genes that comprise the human genome and physically characterize the sites of chromosomal alterations (copy number variations [CNVs], inversions, translocations) in the human population, associated with the predisposition to different diseases, including cancer. It is our belief that such a library and analysis of the human genome will be of great importance to the growing field of gene therapy, new drug design methods, and genomic research. In this review, we detail the motivation for TAR cloning for human genome studies, biotechnology, and biomedicine, discuss the recent progress of some TAR-based projects, and describe how TAR technology in combination with HAC (human artificial chromosome)-based and CRISPR-based technologies may contribute in the future.

## Main Text

The ability to quickly and accurately isolate full-length genes, including coding (exons) as well as non-coding regions (introns and up- and downregulators) that participate in controlling gene expression, has become especially relevant in recent years in light of the growing interest in gene function studies and gene therapy. Gene therapy is a technique for correcting defective genes responsible for disease development.^1^, ^2^, ^3^, ^4^ Researchers identify a defect in a gene that is responsible for a disease and attempt to replace the defective gene with a healthy one, either by using viral and non-viral approaches that are based on expression of full-length cDNA in gene-deficient cells^5^ or by repairing the defect using CRISPR/Cas9 genome editing systems.^6^ The field of genetics is also becoming increasingly important in drug design for personalized medicine. Being able to monitor the effect of a drug treatment on a genetic level greatly enhances our understanding of how a drug affects the patient. When the effect of the drug is better understood, this knowledge can be used to create more effective versions of that treatment and to create variants of the drug that are more effective for specific groups of patients.^7^, ^8^ The difficulty of isolating and manipulating gene variants has been a great hindrance to these areas of research.

**Figure 1:**
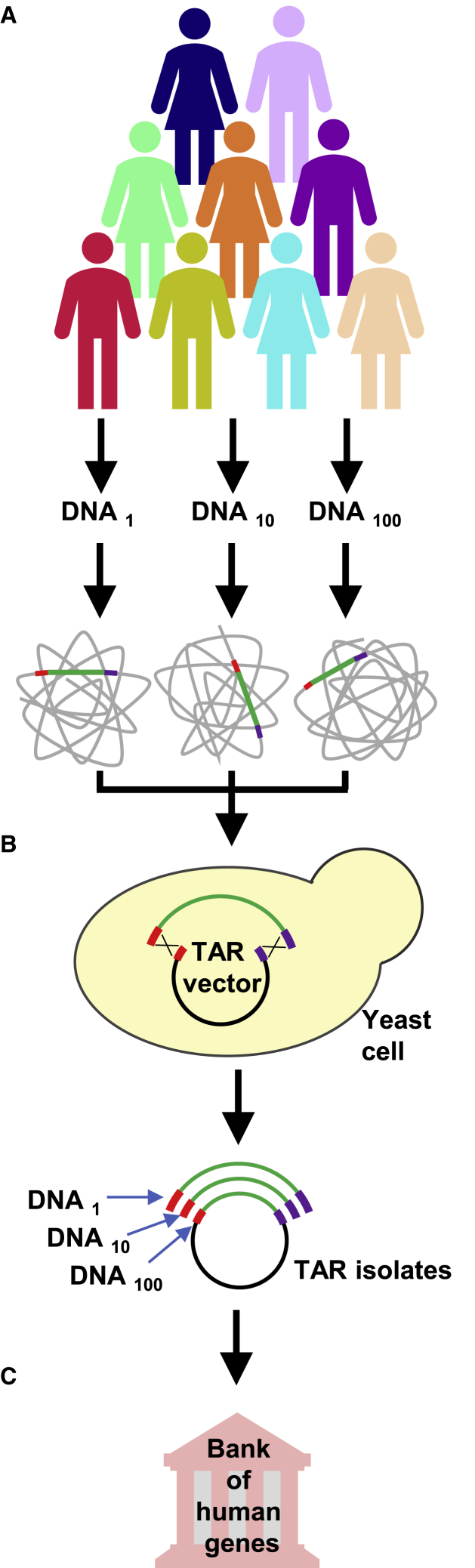
Isolation of a Specific Region or Gene from Multiple Human Genomes by TAR Cloning in Yeast *S. cerevisiae* (A) The TAR cloning procedure includes preparation of yeast spheroplasts and transformation of the spheroplasts by gently isolated high-molecular-weight total genomic DNA along with a TAR vector containing the targeting sequences (hooks). The hooks can be as small as 60 bp.^111^ (B) For TAR cloning experiments, the vector DNA is linearized by a unique endonuclease located between the hooks to expose targeting sequences. Recombination between the vector hooks and homologous sequences in the co-transformed human DNA fragment results in rescue of the desired region as a circular yeast artificial chromosome (YAC) that replicates and segregates properly.^11^, ^12^, ^13^, ^14^, ^15^, ^16^, ^17^, ^18^, ^19^, ^20^, ^21^, ^22^, ^23^, ^24^, ^25^, ^26^, ^27^, ^28^, ^29^, ^30^, ^31^, ^32^, ^33^, ^34^, ^35^, ^36^, ^37^, ^38^, ^39^, ^40^, ^41^, ^42^ The same TAR vector can be applied to multiple DNA samples. If genomic DNA is pre-treated with CRISPR/Cas9 nucleases to generate double-strand breaks near the targeted genomic region, this results in a dramatic increase in the fraction of gene-positive colonies (up to 32 times).^28^ Because the TAR vector contains a BAC cassette, a TAR-isolated YAC clone containing a gene or genomic fragment of interest can be easily moved to bacterial cells by electroporation. (C) Using a set of TAR vectors with different hooks, TAR cloning may help construct a bank of full-length human genes ready to be transferred into human cells for gene function studies and, potentially, for gene therapy.

The transformation-associated recombination (TAR) cloning approach allows entire genes and large chromosomal regions to be selectively and accurately isolated from total genomic DNA.^9^, ^10^, ^11^, ^12^, ^13^, ^14^, ^15^, ^16^, ^17^, ^18^, ^19^, ^20^, ^21^, ^22^, ^23^, ^24^, ^25^, ^26^, ^27^ The method exploits a high level of recombination between homologous DNA sequences during transformation in the yeast *S. cerevisiae*. Figure 1 illustrates TAR isolation of a gene or locus from a genome. Recombination between the homologous sequences from a genome and targeting sequences from the TAR vector leads to establishment of a circular molecule in a YAC (yeast artificial chromosome) form containing the desired genomic fragment carrying a gene or locus. When TAR cloning is applied to complex genomes, such as the human genome, the technique produces YAC clones containing the desired genomic fragment at a frequency of 0.5%–2% of all yeast colonies screened. With the optimized protocol, which includes pre-treatment of genomic DNA with CRISPR/Cas9 nucleases to generate double-strand breaks near the targeted genomic region, the yield of gene- or region-positive clones may be as high as 32%.^28^ Because the same TAR vector can be simultaneously applied to DNA samples isolated from multiple individuals, the TAR cloning approach may be used to generate a bank of gene variants from different population groups (Figure 1) for gene functional studies and for mutational analysis of the same gene using DNA samples from patients with different diseases.

At present, TAR cloning has become a valuable procedure for manipulation of large DNA molecules. The ability to selectively isolate genomic regions and genes has greatly advanced structural and functional analysis of complex and simple genomes and also has different practical applications for biomedicine and biotechnology.^27^ Over the past years, TAR cloning has been used to isolate dozens of unique regions and full-length genes from the genomes of humans, African great apes, the mouse, and other organisms (Table 1).^11^, ^12^, ^13^, ^14^, ^15^, ^18^, ^19^, ^20^, ^21^, ^22^, ^23^, ^29^, ^30^, ^31^, ^32^. Application of the TAR cloning strategy has been demonstrated for long-range haplotyping,^33^, ^34^, ^35^, ^36^, ^37^ and, as a result, this technique is well suited for large-scale analysis of haplotypes in multiple heterozygous individuals to identify haplotypes that might contribute to disease. Because up to 15% divergence in DNA sequences does not prevent cloning in yeast,^38^ TAR technology is a powerful tool for comparative genomics and evolutionary studies. Many gene homologs from nonhuman primates have been TAR-isolated using available human sequence information for targeting.^29^, ^30^, ^31^, ^32^ TAR cloning may be put to use in the final phase of complex genome sequencing and significantly contribute to closing the gaps and verifying the accuracy of contig assembly.^39^, ^40^, ^41^, ^42^ Many genes in the human genome reside within segmental duplications (SDs) with a high level of similarity, which prevents their mutational analysis by routine PCR methods. A TAR cloning strategy has been successfully applied to recover and analyze several duplicated regions, and, therefore, this approach is applicable to all gene families whose analysis is prohibited by the presence of SDs.^29^, ^32^, ^34^, ^35^, ^36^ Accumulating information shows a plethora of structural variations (such as deletions, insertions, inversions, translocations, and copy number variations [CNVs]) in genomes of different individuals, some of which may lead to disease.^43^, ^44^, ^45^, ^46^, ^47^ TAR cloning thus becomes a unique tool to discover and characterize the structural variations causing Mendelian disorders.^35^, ^36^, ^37^ This information may be valuable for diagnostics as well as for the development of new drugs. As seen from the past decade, the cost, quality, and efficacy of the TAR cloning method has been improved significantly by rigorous and comprehensive testing for the accuracy of locus isolation from different organisms, shifting the technology from being a luxury to becoming essential and routine.

**Table 1:** Examples of Chromosomal Regions and Genes or Gene Clusters Isolated by TAR Cloning

Gene or Region	Source	Location	Size (kb)
*BRCA2*	human	13ql3.1	100
*BORIS*	human	20ql3.31	80
*TERT*	human	5pl5.33	150
*SPANX-D*	human	Xq27.2	107
*PTEN*	human	10q23.31	110
*I’M.*	human	3p25.3	50
*NBS1*	human	8q21.3	60
*PKD1*	human	16pl3.3	74
Locus *CAD*	human	9p21.3	230
*KAI1*	human	llpll.2	180
Gapl	human	19pl3.1	90^a^
Gap 2	human	19pl3.2	120^a^
Gap3	human	19pl3.3	180^a^
Gap6	human	19pl3.4	100^a^
*HPRT*	human	Xq26.1	250
*BRCA1*	human	17q21.31	84^b^
	chimpanzee	17	84
	gorilla	nd	66
	orangutan	nd	65
	rhesus macaque	nd	65
*ASPM*	human	lq31	65^b^
	chimpanzee	1	65
	gorilla	nd	65
	orangutan	nd	65
	rhesus macaque	nd	65
*SPANX-C*	human	Xq27.2	83^b^
	chimpanzee	X	58
	gorilla	nd	61
	bonobo	nd	58
*SPANX-B*	human	Xq27.1	90^b^
	chimpanzee	X	63
	gorilla	nd	63
*NBS1*	human	8q21.3	90^b^
	chimpanzee	8	60
	bonobo	nd	60
*ATM*	human	1lq22	150^b^
	rhesus macaque	nd	140
*Hpii*	mouse	X	200
*Peg3*	mouse	7	180
*Muc2*	mouse	7	100
Locus *RNR4*	human	21.pl2	820^c^
Xq27	human	Xq27.1-3	750^d^
Gene cluster	*Saccharomonospora* sp.		67
Gene cluster	*Salinispora pacifica*		21
Gene cluster	*Bacillus subtilis*		17
Gene cluster	*Pseudoalteromonas piscicida*		34
Gene cluster	*B. subtilis*		38
Gene cluster	*S. albus*		36
Gene cluster	*S. aureofaciens*		44
Gene cluster	*S. sclerotialus*		33

nd, not determined.

a Sequences missing on the chromosome 19 were TAR-cloned, sequenced, and deposited into GenBank.^39^

b The same regions or genes were cloned from different species using TAR vectors containing human-specific targeting sequences developed from 5′ and 3′ gene-flanking regions.^29^, ^30^, ^31^, ^32^

c Multiple clones carrying human rDNA units with sizes up to 140 kb were isolated from chromosome 21. Accurate long-read sequencing of 13 TAR isolates of rDNA units covers ∼0.82 Mb of the chromosome 21 rDNA cluster.^42^

d Direct isolation of a set of overlapping 50- to 120-kb genomic segments by *in vivo* recombination in yeast was used to perform a mutational analysis of the 750-kb region in X-linked families. These results excluded the 750-kb genetically unstable region at Xq27 as a candidate locus for prostate malignancy.^36^

### TAR Cloning to Provide a Collection of Full-Length Human Genes

At present, the science of genomes has moved from a phase of genome sequencing into a phase of analysis of gene expression and function. Conventional gene therapy has some success in curative treatment for some primary immune deficiencies in the last decade because of advances in genome editing.^48^ More specifically, development of the CRISPR system created the opportunity to site-specifically correct mutated DNA base pairs or insert a corrective cDNA minigene while maintaining gene expression under the control of endogenous regulatory elements.^6^, ^49^, ^50^

Nevertheless, although cDNA clones are still widely used for gene expression studies, the availability of clones containing full-length genes with all of the necessary regulatory regions, including their own promoter and intronic sequences, would provide indisputable advantages because only in such a configuration can “physiological” expression be achieved. Until recently, the only sources of full-length genes were clones from BAC (bacterial artificial chromosome) and YAC libraries. However, construction of gene expression cassettes using BAC or YAC clones, if found, is time consuming and hit or miss because a gene is often available as a set of fragments in different clones that must be laboriously pieced together. Even if a yeast or BAC contains an entire gene, its use for expression experiments requires trimming of the insert to remove unrelated flanking sequences, often including extraneous genes. In contrast, TAR cloning technology is selective and efficient and allows high-fidelity isolation of full-length genes with all of their regulatory elements (in these cases, the TAR-isolated human genes and their homologs were sequenced).^19^, ^22^, ^27^, ^29^, ^30^, ^31^ In perspective, TAR cloning engineering may provide a complete collection of full-length human genes (Figure 1) ready for CRISPR-mediated insertion or replacement with the following gene expression studies to elucidate the biological function of the disease-associated genes and to help with gene therapy for disease treatment.

### TAR Cloning to Study the Genetic Basis of Human Disease

**Figure 2:**
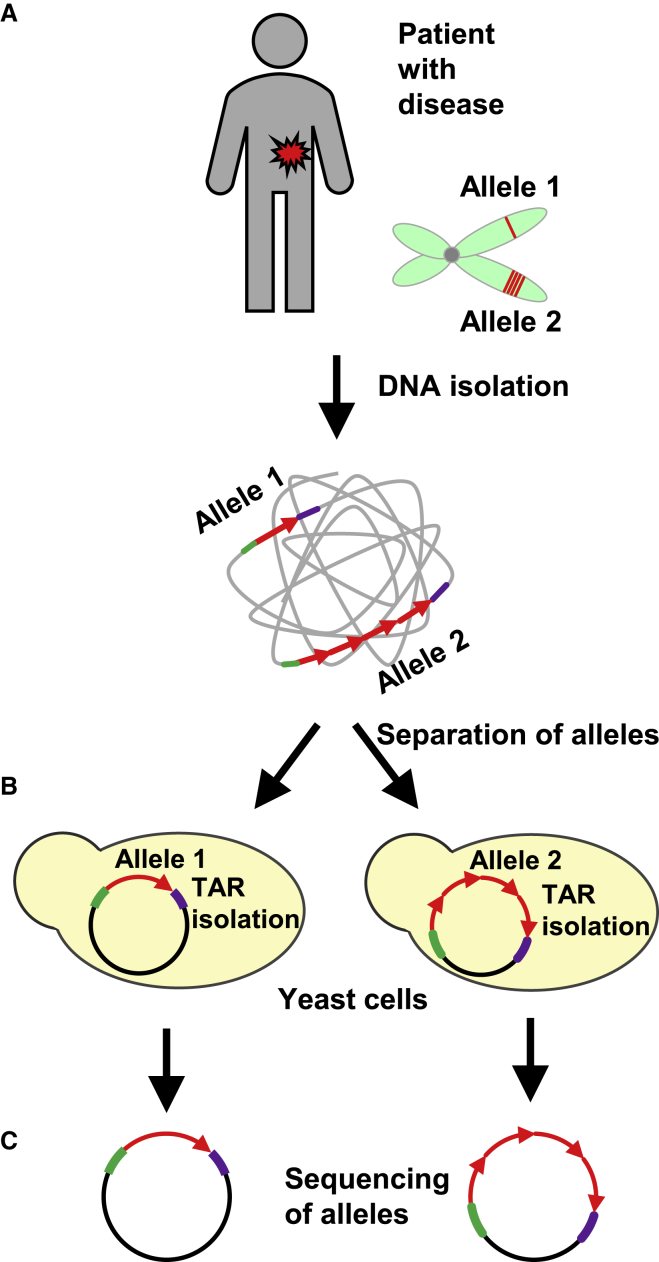
Separation of Parental Alleles by TAR Cloning (A) TAR cloning may be employed to isolate parental alleles of any gene from a patient^33^, ^34^, ^35^, ^36^, ^37^ or a normal individual and determine the specific mutations or rearrangements at polymorphic sites of each allele. The diagram shows separation and isolation of two alleles, allele 1 (normal) and allele 2 (mutant), from the unique genomic region of a patient. (B) Yeast spheroplasts are transformed with genomic DNA isolated from the patient, along with a linearized TAR cloning vector containing 5′ and 3′ unique sequences (green and in purple) specific to the targeted region (red). (C) After TAR cloning in yeast and transfer of TAR isolates to bacterial cells, BAC DNAs are sequenced to identify rearrangements/mutations specific to an allele.

It is well known that hereditary genomic alterations frequently underlie genetic predisposition to disease in humans, including the initiation and progression of cancer. Genomic loss or amplification, translocations, and inversions in many specific regions show consistent association with certain diseases, indicating that certain regions harbor key disease-associated alterations.^28^, ^29^, ^31^, ^32^, ^38^ Importantly, the vast majority of such alterations remain uncharacterized, in part because of technical hurdles. Systematic identification of these chromosomal rearrangements and their cloning and characterization at the molecular level are increasingly needed to obtain a comprehensive description of the genomic alterations leading to cancer or age-related disease. Such an effort would provide the most important and general foundation for basic, clinical, and commercial work in the future. Comprehensive identification of genomic alterations predisposing to disorders requires examination of a substantial number of human individuals. In principle, third-generation sequencing technologies, such as the Oxford Nanopore producing reads up to 700 kb, could be applied for this study.^51^, ^52^ However, wide use of long-read sequencing is limited by its high error rate (approximately 2 of 10 bases).^51^, ^52^ Thus, achieving this ambitious goal is within reach by using TAR cloning technology, which provides a unique tool to rapidly isolate the same region or gene with a certain allele^33^, ^34^, ^35^, ^36^, ^37^ (Figure 2) from hundreds of individuals or patients to identify and characterize a specific chromosomal variation linked to disease. A good example of the application of TAR cloning for verification of gene disease mapping is the analysis of the Xq27-q28 region. Previously, several linkage studies have provided evidence for the presence of the hereditary prostate cancer locus, HPCX1, at Xq27-q28.^53^ The strongest linkage peak for prostate cancer overlied a variable region of ∼750 kb at Xq27 enriched by large SDs, suggesting that predisposition to prostate cancer may be a genomic disorder caused by recombinational interaction between SDs. Direct isolation of a set of overlapping genomic segments carrying SDs completely covering the 750-kb region by TAR cloning was used to perform a mutational analysis of this region in patients. The subsequent analysis of these TAR-isolated fragments excluded the 750-kb genetically unstable region at Xq27 as a candidate locus for prostate malignancy.^36^ The polymorphism information accumulated during analysis of this region may be useful for the analysis of other disease-associated loci that were also mapped to Xq27-q28, including the TGCT locus linked to hereditary testicular cancer and the susceptibility loci for dyslexia, autism, and migraine.^54^, ^55^ In the future, the accumulated information from individual genomes can help predict the pharmaceutical drug response for each individual and expand the therapeutic window of a treatment. In addition, the uniqueness of the technology will help with the design of diagnostics for genomic disorders caused by chromosomal rearrangements.

### TAR Cloning to Illuminate Genomic Dark Matter

The recently reported human genome sequence (hg38) still includes some gaps. For example, five acrocentric chromosomes contain gaps corresponding to clusters of rRNA (rDNA) genes. Despite the key role of the human ribosome in protein biosynthesis, little is known about the extent of rDNA sequence variation within one chromosome and between chromosomes. Prior attempts to characterize rDNA tandem repeat regions have failed because of their highly repetitive structure. Recently, human chromosome 21 rDNA segments were recovered using TAR cloning technology.^42^ Analysis of these TAR-isolated clones revealed previously missed palindromic structures as well as hundreds of variant alleles in the 45S transcription unit and in the non-transcribed spacer. The large number of variants reveals more—and more universal—heterogeneity in human rDNA than considered previously, opening the possibility of corresponding variations in ribosome dynamics. The variants can be assessed for any genetic associations, and the TAR clones provide reagents for further study of human nucleolar assembly and activity. Different variants of human rDNA units may help to clarify the peculiarity of PolI transcription machinery as well as the mechanism by which rDNA units are selected and targeted for chromatin changes leading to heterochromatinization in nucleolar organizer regions (NORs). It is interesting that some variants of a coding region of 21 rDNA units recovered by TAR cloning are located in segments that are conserved between human and yeast.^26^ Functional analysis of these variants *in vivo* (i.e., in the presence of many copies of *rRNA* genes in the chromosomal rDNA repeats) is possible using the yeast rDNA assay developed by Chernoff et al.^56^ Thus, sequence variants identified in 18S and 28S regions that are conserved between human and yeast can be analyzed. In the future, TAR cloning may be applied to cover the rDNA gaps on other chromosomes.

Knowledge of rDNA function is very important because of the dosage effects of human ribosomal genes (rDNA) in health and disease.^57^ rRNA gene dosage affects normal growth and aging, stress resistance of healthy individuals, and survivability of patients with chromosomal abnormalities as well as the risk and severity of some multifactorial diseases with proven genetic predisposition. A study showed that patients with acute systemic purulent infection who had developed multiple organ failure had a low genomic dosage of active ribosomal genes.^58^ The authors concluded that the pattern of purulent inflammation is more severe in subjects with low genomic dosages of active ribosomal genes. Reduced ribosomal activity has been suggested to be a major contributor to the pathology of Alzheimer’s disease (AD).^59^ The rDNA copy numbers were also higher in SZ (schizophrenia) patients.^60^ The presence of palindromic structures as well as certain variant alleles in the 45S transcription may also contribute to human diseases. Thus, TAR closing of gaps corresponding to clusters of rRNA genes and their subsequent analysis is very important for elucidating the source of and predisposition to many diseases.

### TAR-Isolated Genes Expressed from HAC-based Delivery Vectors for Gene Function Studies with a Potential for Gene Therapy

Despite progress with the CRISPR system, which is regarded as an eminent genome engineering tool in therapeutics,^6^, ^50^ we still believe that further progress in functional genomics and gene therapy depends on the availability of both full-length genes and a suitable system for gene delivery and gene expression. The most promising vector system is now human artificial chromosomes (HACs).^61^, ^62^, ^63^, ^64^, ^65^, ^66^, ^67^, ^68^, ^69^ HACs represent extra chromosomes carrying all of the required components of a functional kinetochore. HACs have advantages as gene expression vectors with potential use in gene therapy. They are stably maintained at a low copy in the host nucleus. Furthermore, they contain no viral genes or proteins and, therefore, should not cause severe immunogenic responses, which have been found to be a serious problem with adenoviral vectors.^61^, ^62^, ^63^, ^64^, ^65^, ^66^, ^67^, ^68^, ^69^ HACs are thus intrinsically safer than traditional viral gene therapy systems. They also have a large genetic packaging capacity, up to several megabases, that is suitable for carrying intact mammalian genes in the context of all of their long-range controlling elements that should confer physiological levels of fully regulated gene expression. Existing strategies for the creation of HACs can be broadly divided into two classes: top down, based on truncation of an existing chromosome into a much smaller mini-chromosome suitable for further manipulation^63^, ^64^, and bottom up, where defined, cloned chromosomal elements (alphoid or alpha-satellite units) are TAR-assembled into a prefabricated molecule^70^, ^71^ that is capable of nucleating formation of a HAC *de novo* upon its introduction into human cells.^70^, ^72^ Several laboratories have demonstrated the efficacy of HACs for delivery and expression of full-length genes up to megabase size *in vitro* (human cell lines) and *in vivo* (mice).^73^, ^74^, ^75^, ^76^, ^77^, ^78^, ^79^, ^80^, ^81^, ^82^

**Figure 3:**
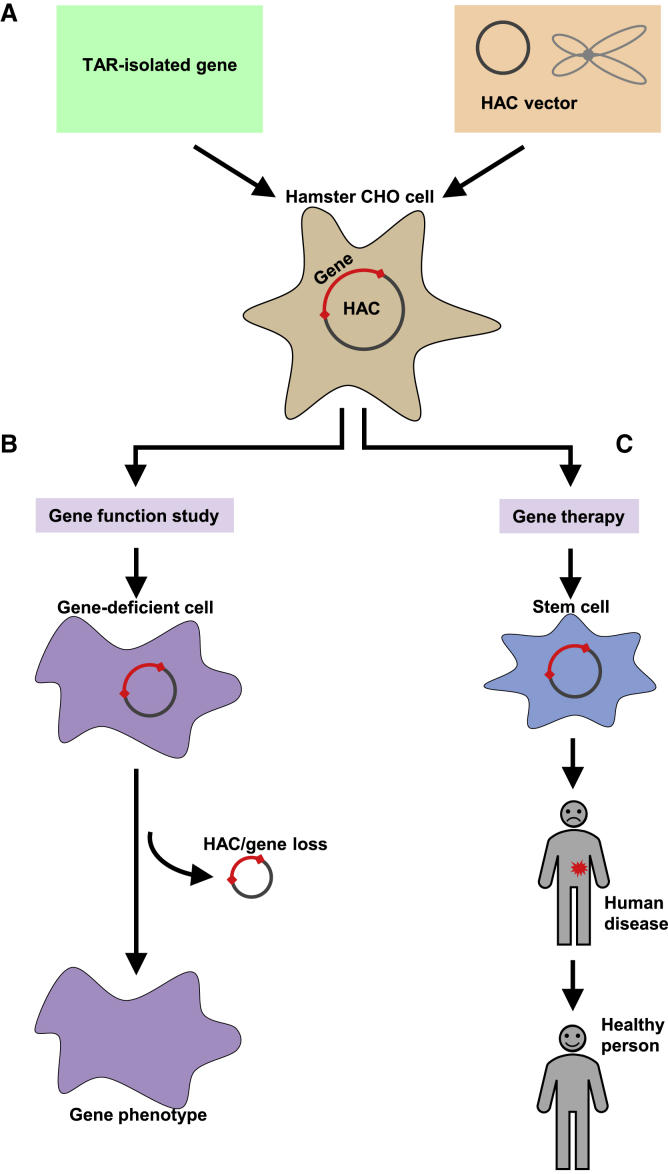
Schematic Diagram Illustrating the Possible Spectrum of Applications of the HAC-Based Gene Delivery Vector Carrying a TAR-Isolated Human Gene for Pluripotent Stem Cell-Based Regenerative Medicine and Gene Function Studies (A) A full-length TAR-isolated gene (green box) is loaded into the HAC-based vector via the Cre/loxP-mediated recombination in hypoxanthine phosphoribosyltransferase (HPRT)-deficient Chinese hamster ovary (CHO) cells. (B) The HAC, along with a gene of interest, is then delivered to target cells (for example, mouse embryonic stem cells [ESCs] or patient-derived human cells deficient for this gene) via an improved microcell-mediated chromosome transfer (MMCT) procedure.^112^, ^113^, ^114^ Thus, the HAC containing a gene can be utilized for functional analyses *in vitro* and *in vivo*, including a humanized mouse model. For the HAC with a regulated kinetochore, the phenotypes arising from stable gene expression from the HAC can be reversed when the HAC is eliminated from the cells by inactivating its kinetochore, which provides control over the phenotypic changes attributed to expression of HAC-encoded genes. (C) For gene therapy, a HAC containing a therapeutic gene can be utilized for treatment of patients with genetic disorders. As a first step, induced pluripotent stem cells (iPSCs) should be produced from patient fibroblasts^114^ using one of the currently available protocols. Then a HAC carrying a therapeutic gene is introduced into iPSCs^115^, ^116^ from donor CHO cells using MMCT. The final step would include transplantation of stem cells into the patient.

At present, HAC vectors are optimized for targeting the genomic regions isolated by TAR technology: (1) gene expression from the HAC-based vector is protected by chromatin insulators,^83^ and (2) an unlimited number of genomic DNA fragments or genes may be assembled on the HAC molecule.^84^, ^85^, ^86^ Combining engineered human chromosomes with TAR cloning technology can greatly advance gene function studies (Figure 3).^61^, ^62^, ^65^, ^73^, ^74^ Moreover, the combination of large-capacity vectors carrying TAR-isolated genes with stem or progenitor cells establishes a novel platform for gene therapy.

### TAR Cloning of Natural Product Biosynthetic Gene Clusters for Biotechnology

**Figure 4:**
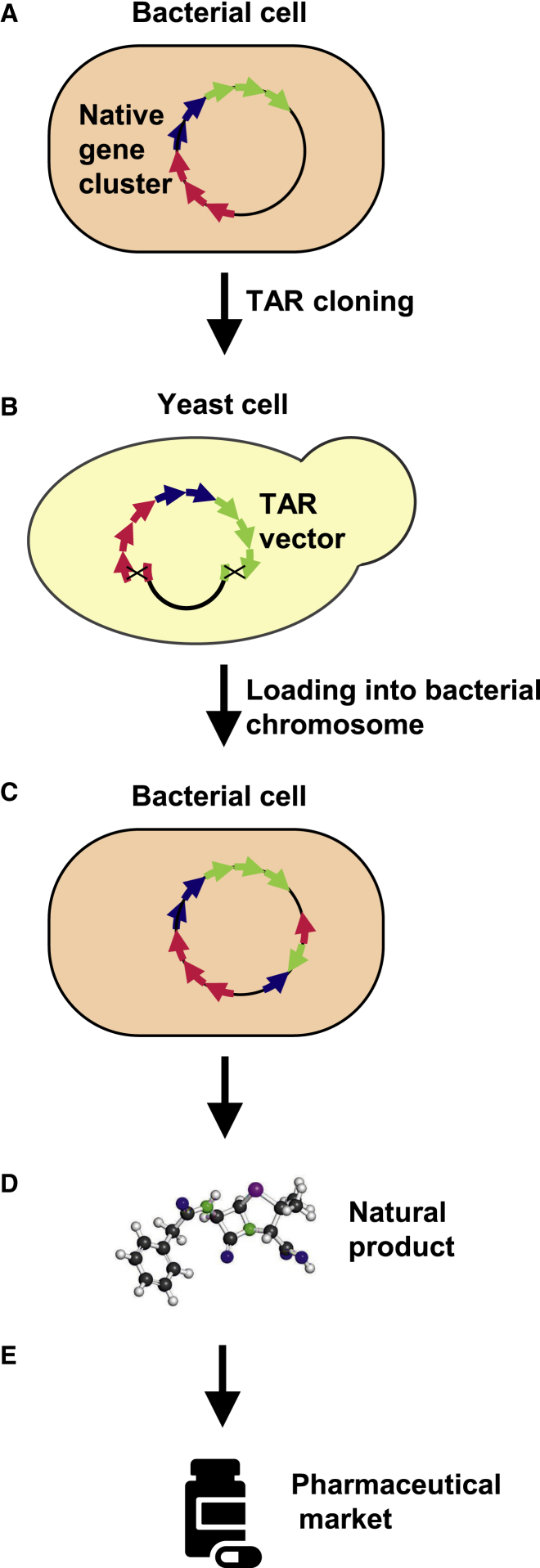
Design of TAR Cloning of Large Natural Product Biosynthetic Gene Clusters from Simple Genomes (A) Physical map of a gene cluster located on a chromosome propagated in a bacterial cell. In a TAR vector, the hooks or homology arms correspond to both ends of the cluster. (B) TAR-based natural product discovery involves homologous recombination between the linearized pathway capture TAR vector and co-transformed genomic DNA isolated from host bacterial cells that leads to formation of a circular YAC/BAC construct. (C and D) Some TAR-cloned natural genes or gene clusters may be transferred to a bacterial host strain and, if necessary, integrated into the chromosome of a host strain for basic research or production of natural compounds. (E) Natural products have provided considerable value to the pharmaceutical industry over the past half-century.

Over the past years, there have been many examples of direct capture of genes or gene clusters by TAR cloning from simple genomes; for example, recovery of large natural product (NP) biosynthetic gene clusters (Figures 4A and 4B).^87^, ^88^, ^89^, ^90^, ^91^, ^92^, ^93^, ^94^, ^95^, ^96^, ^97^, ^98^, ^99^, ^100^, ^101^ So far, TAR cloning has been applied for isolation of genomic regions and genes up to ∼250 kb (Table 1). Therefore, the TAR method has an advantage, especially in the case of NP biosynthetic gene clusters in microbes, whose size is much smaller. The TAR-cloned natural genes or gene clusters may be transferred from yeast to a bacterial host strain as BACs-YACs or, if necessary, integrated into the chromosome of a host strain for basic research or production of natural compounds (Figures 4C and 4D). More specifically, the lipopeptide gene cluster from the marine actinomycete *Saccharomonospora* sp., which yields the antibiotic taromycin A,^89^ the *Salinispora pacifica* NP gene cluster for biosynthesis of enterocin,^90^ and the amicoumacin biosynthetic gene cluster from the marine *Bacillus subtilis 1779* for antibiotic production in the *Bacillus* host^91^ were selectively TAR isolated. NPs and their derivatives contribute to a huge pharmaceutical market share comprising 61% of anticancer drugs and 49% of anti-infection medicine in the past 30 years^102^.

It is worth noting that TAR cloning requires that the cloned DNA fragment carries at least one autonomously replicating sequence (ARS) that can function as the origin of replication in yeast. Sequences that function as ARSs in yeast occur, on average, every 20–40 kb in all of the eukaryotic genomes examined so far,^13^, ^17^ which suggests that most mammalian chromosomal regions can be readily isolated by TAR cloning with a vector that lacks an ARS. The ARS frequency in some microbe genomes may be reduced, meaning some regions may not be isolatable by the original TAR method. Therefore, we developed a general TAR cloning system for isolation of genomic regions regardless of the presence of ARS elements.^103^ In this system, the TAR vector contains an ARS and a counter-selectable marker. Negative genetic selection eliminates the background because of vector recircularization caused by end joining during yeast transformation. Recently, a similar, very effective TAR capture from microbe genomes was developed by another lab^98^ that also used a counter-selectable marker to improve efficiency of cloning. Both methods are powerful tools for structural and functional analysis of prokaryotic genomes.

Isolation and manipulation of NP biosynthetic gene clusters have further accelerated our understanding of their molecular biosynthetic mechanisms, leading to a rational redesign of novel NPs.^104^ To summarize, TAR cloning of gene clusters from microbe genomes provides an opportunity to address fundamental problems in environmental evolution and biotechnology.

### Conclusions

TAR engineering may physically characterize all of the sites of chromosomal alterations in the human genome associated with predisposition to different diseases, including cancer. Accumulated comprehensive knowledge of the genetic basis of these diseases will provide a foundation for future medical research and have far-reaching implications for basic, clinical, and commercial efforts to understand, prevent, and treat diseases and develop new strategies for their diagnosis and treatment. TAR technology may become a foundation for creating a collection of annotated human genes, each of which is represented by a genomic copy containing coding regions (exons), 5′ upstream and 3′ downstream regulatory sequences, and introns. The availability of full-length genes with their own intact regulatory elements will catalyze major breakthroughs in functional, structural, and comparative genomics; diagnostics; gene replacement; and generation of animal models for human diseases, with a potential for gene therapy from which a new generation of genomic research products could be launched. Notably, the large size of genes or gene clusters does not prevent their propagated in yeast based on TAR assembly of whole-microbe genomes with sizes greater than 1.5 Mb.^105^, ^106^, ^107^ This unique technology may be used in combination with HAC-based gene delivery vectors for stable and regulated production of therapeutic proteins through regulated expression of full-length human genes. In addition, these technologies should improve the success rate of drug development related to efficacy and safety for humans.^108^ In perspective, given the potential of engineered human chromosomes of curing genetic defects, HACs offer tremendous potential for gene therapy applications. Development of a universal gene delivery system for conventional gene therapy and for stable and regulated production of therapeutic proteins may be an ambitious goal in the near future.

**Figure 5:**
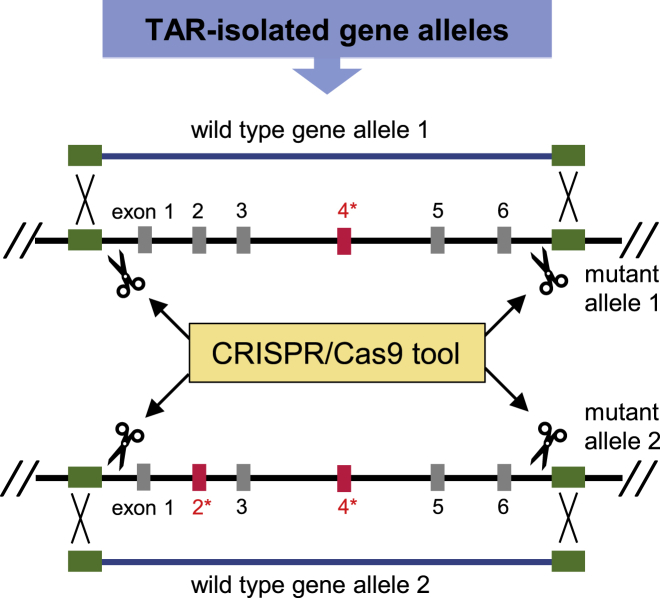
Schematic of a CRISPR/Cas9-Based Strategy to Simultaneously and Differentially Replace Two Mutant Gene Alleles of a Diploid Cell by Two TAR-Cloned Wild-Type Gene Alleles in a Single Step A gene of interest is targeted with two single guide RNA (sgRNAs; green rectangles) at the 5′ and 3′ ends of the gene. Two repair templates are introduced into the cell, which mediate the replacement of the gene on both alleles. Mutant exons are marked in red.

For the past decade, CRISPR/Cas9-based methodologies have promise to lead to rapid manipulation of endogenous genes that will open the door for new synthetic biology platforms. Recently, a new gene replacement strategy utilizing CRISPR/Cas9 technology was devised.^109^, ^110^ In this strategy, a mutant gene in the human genome is simultaneously and differentially replaced on both alleles. CRISP/Cas9 technology combined with TAR-cloned gene alleles may be applied for gene functional analysis with perspectives on gene therapy (Figure 5). When such an approach is optimized, the study of complex biological systems will become more powerful and convincing for the investigator. In future, TAR-based gene technology may be used to carry out a regular customer service for isolation of genomic regions and entire loci from clinical materials and alternative genomes (Table 1).

## Conflicts of Interest

The authors declare no competing interests.
